# Enhancing the quality of fermented plant leaves: the role of metabolite signatures and associated fungi

**DOI:** 10.3389/fpls.2024.1335850

**Published:** 2024-03-20

**Authors:** Lei Xing, Jinshan Lei, Jie Liu, Zhen Yang, Zhishun Chai, Wen Cai, Min Zhang, Delong Meng, Yujie Wang, Huaqun Yin

**Affiliations:** ^1^ China Tobacco Sichuan Industrial Co., Ltd, Chengdu, China; ^2^ Cigar Fermentation Technology Key Laboratory of China Tobacco (China Tobacco Sichuan Industrial Co., Ltd.), Chengdu, China; ^3^ Industrial Efficient Utilization of Domestic Cigar Tobacco Key Laboratory of Sichuan Province, Chengdu, China; ^4^ School of Minerals Processing and Bioengineering, Central South University, Changsha, China; ^5^ Key Laboratory of Biometallurgy, Ministry of Education, Changsha, China

**Keywords:** fermentation, fungal, metabolites, interactions, plant leaves

## Abstract

Fungi play a pivotal role in fermentation processes, influencing the breakdown and transformation of metabolites. However, studies focusing on the effects of fungal–metabolite correlations on leaf fermentation quality enhancement are limited. This study investigated specific metabolites and fungi associated with high- and low-quality fermented plant leaves. Their changes were monitored over fermentation periods of 0, 8, 16, and 24 days. The results indicated that organoheterocyclic compounds, lipids, lipid-like molecules, organic nitrogen compounds, phenylpropanoids, and polyketides were predominant in high-quality samples. The fungi *Saccharomyces* (14.8%) and *Thermoascus* (4.6%) were predominantly found in these samples. These markers exhibited significant changes during the 24-day fermentation period. The critical influence of fungal community equilibrium was demonstrated by interspecies interactions (e.g., between *Saccharomyces* and *Eurotium*). A co-occurrence network analysis identified *Saccharomyces* as the primary contributor to high-quality samples. These markers collectively enhance the quality and sensory characteristics of the final product.

## Highlights

- Specific metabolites and fungi were significantly correlated with plant leaf quality.
- *Saccharomyces* was the main driver of plant leaf quality during fermentation.
- *Eurotium* was indispensable in regulating the metabolic features of plant leaves.

## Introduction

Plant leaf fermentation is increasingly recognized as an effective method for enhancing quality and introducing unique characteristics. Environmental factors, including optimal temperature, humidity, and moisture conditions, along with microbial activity, significantly influence quality enhancement during fermentation (Drobek et al., 2019; Zhang et al., 2019; Zhu et al., 2020). For example, fermenting medicinal herbs into Yaoqu can lower toxicity, increase effectiveness, and satisfy the exacting standards of clinical applications (Li L. et al., 2020). Microbial activity can alter the chemical composition of plant leaves throughout the fermentation process (Zhu et al., 2020). Liu F. et al. (2021) demonstrated that the aromatic substance content and quality of tobacco leaves have been significantly influenced by changes in bacterial and fungal communities during fermentation. Previous studies have confirmed the positive effects of metabolites and microorganisms on enhancing the quality of fermented plant leaves (Zeng et al., 2019; Karim et al., 2020; Yu and Yang, 2020; Zhu et al., 2020). However, which microorganisms and metabolites of plant leaves dominated the fermentation processing and how they work together to improve the quality of fermented plant leaves still remain poorly understood. Therefore, deepening our understanding of the variables and mechanisms involved offers a significant potential for advancing plant leaf fermentation techniques.

It is essential to investigate the biochemical changes associated with specific metabolites in plant leaves during fermentation, as these alterations fundamentally determine the quality of the fermented leaves (Li J. et al., 2020; Liao et al., 2022; Wang et al., 2022). Several studies have demonstrated that certain beneficial metabolites can neutralize harmful odors, degrade toxic substances, diminish unpleasant smells, and generate distinct flavors, thereby improving the quality of plant-based products (Liu F. et al., 2021; Jia et al., 2023). A total of 47 compounds, including catechins, flavonoids, and flavoalkaloids, were identified by Cheng et al. (2021) as critical metabolites responsible for the quality of Qingzhuan tea under age-related variations. Yue et al. (2019) found 21 metabolites, including glycoside derivatives, flavonoids, and phenolic acid derivatives, had the potential to serve as markers for distinguishing distinct grades of Bai Mudan white tea.

Fungi can give fermented plant leaves distinct flavors and advantages (Pusztahelyi et al., 2015; Shang et al., 2022). *Aspergillus* and *Bacillus* are the predominant fungi in Qingzhuan tea, while *Aspergillus* species predominate in Pu-erh tea. This leads to notable variations in the teas’ sensory attributes and metabolic aspects (Cheng et al., 2020). Studies have explored the dynamics of fungal communities and metabolites in plant leaves during the fermentation process. The fluctuations in chemical compositions and sensory attributes are motivated by primary fungal species (Ma et al., 2021). *Eurotium* greatly influenced the metabolites, phenolic acids, and flavonoids of dark tea, which provided valuable effects in shaping the unique characteristics of postfermented Fu brick tea (Xiao et al., 2022). Thus, the correlations between fungi and metabolites in plant leaves warrant increased attention during the fermentation process.

It is well established that the composition of specialized metabolites in plant leaves is significantly influenced by a variety of environmental conditions (Neilson et al., 2013; Mierziak et al., 2014; Erb and Kliebenstein, 2020). The chemical composition of plant leaves may be dramatically changed by variations in fermentation conditions (Tang et al., 2018; Li J. et al., 2020). For example, numerous studies have been done to investigate how humidity affects the quality of plant leaves (Hirai et al., 2000; Zhao et al., 2022). According to Zhao et al. (2022), a humidity level of 70% was found to be ideal for enhancing the quality of tobacco leaves used to make cigars. Therefore, we primarily identified the metabolite signatures and associated fungi in high- and low-quality fermented plant leaves. Subsequently, we revealed their changes over time during fermentation at an optimal humidity of 70%.

Fermented plant leaves play a critical role in providing essential nutrition or economic support on a global scale. It is imperative to improve the quality of these products to benefit both producers and consumers. Advancing our understanding of the role of metabolite signatures and fungi in the fermentation process is key to furthering knowledge in microbial ecology and biotechnology, offering potential applications in quality enhancement and flavor innovation during fermentation. In this study, the metabolite composition and the fungal community structure of all samples were systematically analyzed by an integrated metabolomic and ITS analysis approach. A part of the cigar tobacco samples was fermented at 62% humidity for 3 years to confirm the key biomarkers of high-quality fermented samples. The conditions (i.e., humidity and time) of the cigar tobacco fermentation were altered to explore the changes in these key biomarkers during fermentation processing. The correlations between metabolites and fungi during fermentation were revealed by co-occurrence network constriction. Our study aims to deepen our understanding of the quality improvement of plant leaves regulated by specific metabolites and fungi during fermentation.

## Materials and methods

### Sample collection and treatment

The cigar samples were derived from a reliable producer in Sichuan, China, in November 2022. A part of the cigar tobacco samples fermented at 62% humidity for 3 years, which were confirmed as the quality-variable groups [low-quality (L) and high-quality (M)] by sensory quality evaluation. The key biomarkers of high-quality fermented samples were confirmed by metabolic and fungal data analysis. The changes of these key biomarkers were observed in other parts of cigar tobacco samples that were respectively fermented at 75% humidity for 0, 8, 16, and 24 days in four time-series groups (T1, T2, T3, and T4). Overall, six different fermentation groups were set as follows: two quality-variable groups (L and M) and four time-series groups (T1, T2, T3, and T4). Prior to the sensory analysis, the samples were rolled into cigars by skilled rollers. A five-member-trained panel clarified the quality of L and M samples by assessing flavor components and sensory characteristic scales. A 5-point hedonic scale was used to determine the flavor component scales, and a 9-point hedonic scale was used to calculate the sensory characteristic scales. A higher score indicated that the associated index performed better. All panelists agreed after discussing and deciding on the ratings. The results showed that sample M was of greater quality than sample L. The specifics of the sensory assessment were provided in the **Supplementary Material** (**Supplementary Tables S1, S2**).

To obtain representative samples for each group, six groups were chopped and mixed. For nontargeted metabolite analysis, 2 g of representative samples were placed into precooled cryopreservation tubes resistant to extremely low temperatures (−192°C), snap-frozen with liquid nitrogen for 3 to 4 h, and stored at −80°C. For DNA extraction and ITS rDNA sequencing, 12–15 g of representative samples were snap-frozen in liquid nitrogen for 1 h and kept at −80°C.

### Analysis of nontargeted metabolites

Following the extraction of metabolites from plant leaf samples, raw data were collected by metabolomic analysis of the samples through the use of mass spectrometry (MS) in conjunction with liquid chromatography (LC). After preprocessing the raw data, a data matrix was created that could be utilized for additional data analysis. The details about the particular procedure and constraints were in line with those of Lei et al. (2023). Using the Kyoto Encyclopedia of Genes and Genomes (KEGG) database, the metabolites were annotated. The data were normalized by the probability quotient normalization algorithm before analysis. For each sample, the data were normalized using a probability quotient normalization procedure. Quality control (QC) samples were then utilized for batch correction using a robust QC spline. Student’s *t*-tests were performed to examine the *p*-value, and the Benjamini–Hochberg false discovery rate (FDR) was then employed to correct multiple tests for variations in metabolite selection. To find more precise differences between groups, we also used supervised partial least squares-discriminant analysis (PLS-DA) utilizing MetaX variable discriminant analysis statistical methods. The contribution rate of the variations in metabolites in various groups is represented by the variable significance in the projection (VIP) value. To identify the key metabolites differentiating six groups, multiply the fold change (FC) by the average value of all biological repeat quantitative data in the comparison group. These key metabolites were finally identified by meeting certain requirements, including FC ≥ 2 or ≤ 1/2, *p*-value ≤ 0.05, and VIP > 1.

### DNA extraction and ITS sequencing

The Plant Genomic DNA Kit (Isolate Plant DNA Minikit, Tiangen, China) was utilized to extract the whole genomic DNA sample. Using agarose gel electrophoresis and a NanoDrop ND-1000 spectrophotometer (Thermo Fisher Scientific, Waltham, MA, USA), the amount and quality of the recovered DNA were assessed. Primers were sequenced using universal primers ITS1 (5′-GTGARTCATCGAATCTTTG-3′) and ITS2 (5′-TCCTCCGCTTATTGATATGC-3′), and their 5′ ends were barcoded according to the sample. A total of 25 ng of template DNA, 12.5 μL PCR Premix, 2.5 μL of each primer, and PCR-grade water to adjust the volume of 25 µL were used in the PCR reactions. An initiation phase of 30 s at 98°C was followed by 32 cycles of denaturation (10 s at 98°C), annealing (30 s at 54°C), and extension (45 s at 72°C) in the thermocycling process. For 10 min, a final elongation step was carried out at 72°C. Following 2% agarose gel electrophoresis to visualize the PCR products, AMPure XT beads (Beckman Coulter Genomics, Danvers, MA, USA) were used to purify the results. Qubit (Invitrogen, USA) was used for the quantification of the refined PCR products. The PCR products were then purified and ready for sequencing. The Illumina library quantitative kits (Kapa Biosciences, Woburn, MA, USA) and an Agilent 2100 Bioanalyzer (Agilent, USA) were used to assess the qualified PCR products. These results were then pooled and sequenced on an Illumina NovaSeq 6000 (PE250), which was made available by LC-Bio Technology Co., Ltd., Hangzhou, China. The NCBI sequencing Read Archive (SRA) contains the ITS rRNA sequencing data.

### Fungal community analysis

The samples were allocated to the raw reads obtained by the Illumina NovaSeq platform paired-end sequencing based on their distinct barcodes, and the primer sequence and barcode were removed to truncate the reads. Using normal operating protocols, all quality filtering and sequence read processing were carried out on a publicly accessible Galaxy pipeline (http://mem.rcees.ac.cn:8080/) (Zhou et al., 2010). To simply summarize the steps, primer fragments were removed using Cutadapt, and low-quality sequences with a quality score of less than 20 and a window size of 5 were removed using Btrim (Kong, 2011). UCHIME was used to identify and eliminate chimera sequences (Edgar et al., 2011). Sequences with ≥ 97% similarity were assigned to the same operational taxonomic units (OTUs) by using UPARSE. Singleton OTUs were eliminated from further investigation, and each OUT’s representative sequences were classified in the UNITE database.

The microeco package in R (Liu C. et al., 2021) (version 4.1.2) was used for all community analysis and statistical work. Using the Vegan package, principal coordinate analysis (PCoA) based on the Bray–Curtis dissimilarity matrix was used to depict community dissimilarity and ordination plots (Oksanen et al., 2013). The linear discriminant analysis effect size (LEfSe) method (Segata et al., 2011) was employed to identify the significantly different taxa across the six fermentation groups. In order to determine the most noteworthy fungal species and validate their impact on enhancing the quality of fermented plant leaves, a logarithmic LDA score threshold of 4 (*p* < 0.05) was established to identify discriminative characteristics.

### Co-occurrence network constriction

Using CoNet in Cytoscape, the co-occurrence correlation network was constructed. Only the samples that appeared in the rows at least once and had available sample counts of more than 50% were kept for further estimations. To identify fungal interactions, a Spearman’s correlation threshold of 0.6 (*p* < 0.05) was utilized. To evaluate the most prominent significance of fungal species and metabolites, a threshold of 0.9 (*p* < 0.05) was employed. Using cytoHubba (Chin et al., 2014), the node scores were computed based on the fungal interaction network in order to predict important taxa. For constructing a hub network, the top 20 Hubba nodes were chosen based on their Matthews correlation coefficient (MCC) ranking (Zhu, 2020). Each of the nodes in a network has a size that corresponds to the number of connections (i.e., degree). Gephi software was used to visualize all networks (Jacomy et al., 2014).

### Statistical analysis

The variations in the metabolites between the six groups were analyzed by ANOVA. The data were displayed using the Pheatmap (v 1.0.12) package in R (Kolde, 2019) after being normalized using z-scores of various metabolite intensity regions. The Euclidean distances between the main fungal taxa were then determined (Murtagh and Legendre, 2014). Using the linkET (v 0.0.7.4) package in R, the Mantel test was performed to find the correlation between metabolites and fungal communities with significant differences (*p <* 0.05) across six fermentation groups by COR (method = Spearman). Using randomForest (4.7-1.1) in R, we performed a classification random forest (RF) analysis (tree = 2,000) to determine the main statistically important metabolite predictors of *Saccharomyces* relative abundance (Altmann et al., 2010). Using Adobe Illustrator CC 2019 (Adobe Systems Inc., San Francisco, CA, USA), all figures were processed and illustrated.

## Results

### Identification of metabolites in different fermentation

**Figure 1:**
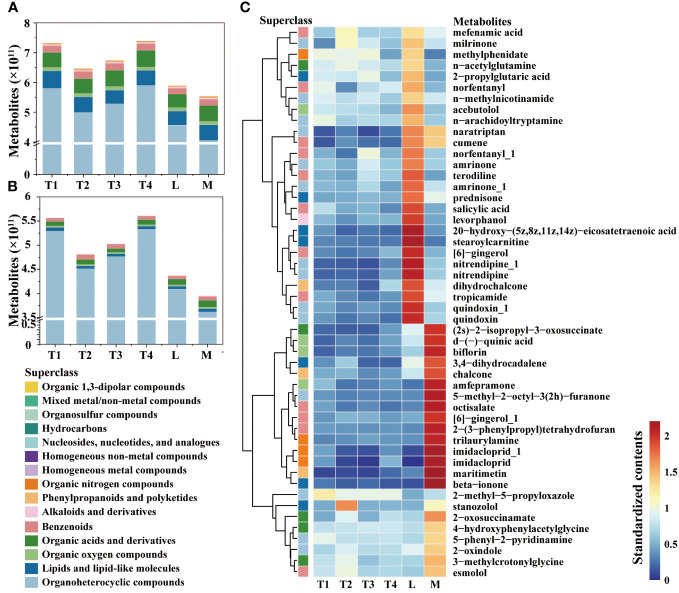
Comparison of metabolites in different fermentation groups. **(A)** The contents of metabolites among six groups. **(B)** The contents of metabolites showed significant differences among the six groups. **(C)** Heatmap and hierarchical cluster analysis based on the contents of metabolites with the top 50 variable importance in projection (VIP).

A total of 8,451 and 2,676 metabolites were annotated for analysis in the positive (POS) and negative (NEG) modes, respectively (**Supplementary Table S3**; **Figure 1A**). These annotated metabolites were classified into 15 superclasses, and 3,460 annotated features exhibited significant differences between the six treatment groups (**Supplementary Figure S1**). Among these superclasses (**Figure 1B**), organoheterocyclic was the most abundant and variable metabolite; the content of that increased along with fermentation days. The contents of homogeneous nonmetal compounds, lipids, lipid-like molecules, organic nitrogen compounds, phenylpropanoids, and polyketides showed significant differences between L and M, which indicated that these superclasses were the main contributors to the high quality of plant leaves. In four series time groups, the contents of these superclasses showed considerable change, which was increased at 8 days (T2) and then decreased at 16 and 24 days (T3 and T4). Conversely, the content of homogeneous non-metal compounds remained relatively stable throughout the fermentation period (0 to 24 days). These results suggest that the first three superclasses should be given special attention at the onset of the fermentation process.

The contents of the top 50 metabolites with significant differences among six groups were compared (**Figure 1C**), and we observed they were enriched in L or M. A total of 15 metabolites were immensely enriched in M group; they were (2s)-2-isopropyl-3-oxosuccinate of organic acids and derivatives, d-(-)-quinic acid, florin and amfepramone of organic oxygen compounds, 3,4-dihydrocadalene and beta-ionone of lipids, lipid-like molecules, chalcone and maritimetin of phenylpropanoids and polyketides, 5-methyl-2-octyl-3(2h)-furanone of organoheterocyclic compounds, octisalate, [6]-gingerol_1 and 2-(3-phenylpropyl) tetrahydrofuran of benzenoids, and trilaurylamine and imidacloprid of organic nitrogen compounds. This distribution of them indicated that the conversion of these metabolites has an indispensable role in quality enhancement. Some metabolites, such as 2-methyl-5-propyloxazole, decreased along fermentation time and were relatively lower in the L and M groups. This trend was similar with its classified superclasses, organoheterocyclic compounds. The dynamics of these metabolites require strict regulation and supervision during the initial fermentation process.

### Comparison of fungal communities

**Figure 2:**
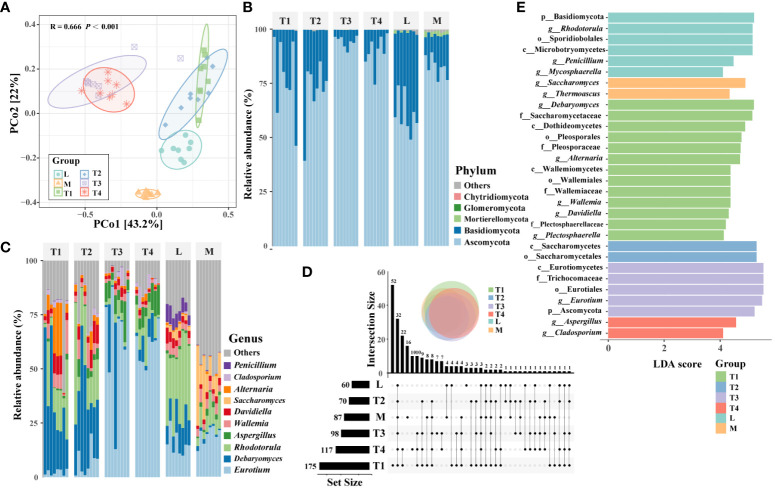
Fungal community composition and distribution traits in six fermentation groups. **(A)** PcoA showing the Bray–Curtis similarities of plant leaf fungal communities. **(B)** An upset diagram illustrating the numbers of shared and unique OTUs among six fermentation groups. The composition of fungal communities in terms of phyla **(C)** and major genera **(D)** among six groups. **(E)** The fungal taxa were identified as biomarkers in six fermentation groups by LEfSe (LDA < 4, *p* < 0.05).

PCoA results showed significant differences (*p* < 0.001, Adonis test) in the structures of plant leaf communities among six groups (**Figure 2A**). The groups of T1 and T2, T3 and T4, were slightly overlapping with each other, respectively, while L and M groups were entirely separate from the other groups, which suggested that the structure of the fungal communities was prominently correlated with the quality of plant leaves. Five phyla were identified in fungal community across all samples, such as Ascomycota and Basidiomycota (**Figure 2B**). These two phyla were the most abundant phyla, accounting for 79.5% and 19.8%, respectively. A comparison of the top 10 genera showed that the dominant genera varied among the six groups (**Figure 2C**). For instance, *Saccharomyces* (14.8%) and *Thermoascus* (4.6%) were most enriched in the M group, which suggested that they were correlated with the high quality of fermented plant leaves. *Alternaria* (10.5%) and *Plectosphaerella* (2.7%) had a higher relative abundance than the other fermented groups; thus, we believed these two genera were easily influenced and reduced by fermentation processing. Noticeably, *Eurotium* (64.6% and 60.6%) was the dominant genera in the T3 and T4 groups, respectively; its relative abundance was significantly higher than other groups, and it was regarded as the main potential factor influencing the quality of plant leaves during fermentation.

The Chao1 and observed OTU indices showed that the alpha-diversity was the highest in the T1 group. This indicated that the unfermented leaves had a higher species than fermented leaves. Conversely, the Shannon and inverse Simpson indices of the M group were significantly (*p* < 0.05) higher than those of the other groups (**Supplementary Figure S2**), which showed that the fermented leaves with high quality had a higher diversity than that of other samples. The number of unique OTUs in the T1 group was the highest, followed by shared OTUs among all six groups (**Figure 2D**). OTU475, OTU345, and OTU356 belonged to *Alternaria*; these three OTUs were the most genera of the 52 unique OTUs in the T1 group. Whereas OTU12, OTU15, OTU29, and OTU894 were part of *Aspergillus*, these four OTUs were the abundant genera of 32 shared OTUs among six groups. These findings indicated that the diversification of fungal communities is significantly influenced by the fermentation process, and certain specific taxa may play a pivotal role in determining the quality of fermented plant leaves.

A total of 30 taxa (LDA > 4, *p* < 0.05) were identified as biomarkers among six groups (**Figure 2E**). The T1 group had the largest number of enriched taxa, which can serve as biomarkers for distinguishing unfermented plant leaves from fermented ones. Five of 13 genera as biomarkers (*Debaryomyces*, *Alternaria*, *Wallemia*, *Davidiella*, and *Plectosphaerella*) were found in the T1 group. The remaining eight genera were distributed more evenly among the L, M, T3, and T4 groups. Specifically, *Saccharomyces* and *Thermoascus* were enriched in high-quality samples of the M- group, which indicated their relative abundance may relate to the high quality of fermented plant leaves. These results suggested that the relative abundance of these eight genera was actively involved in the fermentation process, and some of them can be remarkably correlated with the quality of fermented plant leaves.

### Interaction processes of fungal communities driven by fermentations

To explore potential microbial interaction patterns among six groups, networks of distinct structures and topologies for the communities of plant leaves were constructed. A broad range of edge numbers was 164~5,117, and node numbers were 60~175 among six groups. The network of the L group had the least number of nodes and edges, which indicated that the network was relatively simpler than the other groups. In contrast, the network indices for the T1 group were the highest among the five groups studied. This includes measures such as the average number of neighbors, clustering coefficient heterogeneity, and centralization, as detailed in **Supplementary Table S4**. These results suggested that the complexity of the network in fermented plant leaves could be reduced during the fermentation process and that high-quality fermented leaves were correlated with more complex networks. Interestingly, we observed that the proportions of positive and negative linkages were fairly equal in the L and M groups, whereas more positive linkages were present in the T1 to T4 groups. These findings indicated that maintaining a balance of positive and negative relationships could contribute to a better fermentation effect and improve the quality of fermented leaves.

We also noticed significant differences in the top 20 hub taxa among the six groups. There are more balanced linkages in the hub network of M (102) than in the L group (52). The linkages of the T1 group were the highest, and that was decreasing in 8 days of fermentation time (T1) and increasing in 16 and 24 days (T3 and T4). These findings further proved the more equally positive and negative links with the higher quality of fermented samples. Also, the more complex interactions were found in high-quality and/or unfermented samples.

**Figure 3:**
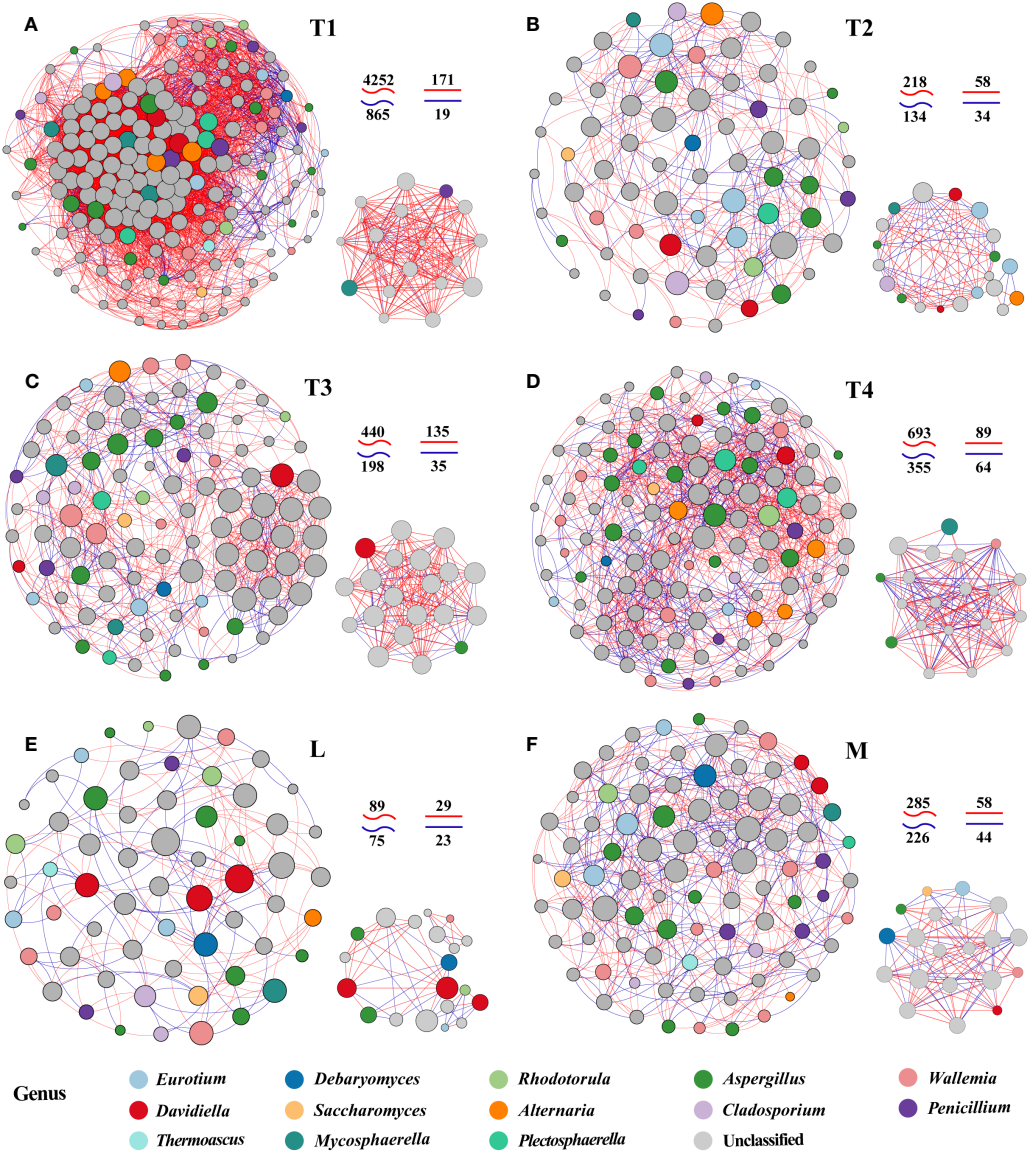
Network patterns of plant leaf communities of L **(A)**, M **(B)**, T1 **(C)**, T2 **(D)**, T3 **(E)**, and T4 **(F)** fermentation groups. A connection indicates a strong (Spearman’s |*r*| >0.6) and significant (*p* < 0.05) correlation. Positive correlations are represented by red lines, whereas negative correlations are represented by blue lines. The size of the nodes corresponds to the degree of operational taxonomic units (OTUs).

Specifically, *Saccharomyces*, identified as one of the six biomarker genera among the 20 hub taxa, was exclusively observed in the M group network. *Saccharomyces* exhibited a positive correlation with *Eurotium* and a negative correlation with *Aspergillus*. *Eurotium* was present in the hub networks of the L, M, and T2 groups, but it rarely appeared in the hub networks of unfermented samples and other fermentation periods. *Aspergillus* was an invariable keystone taxon in all L groups, which highlights its indispensable role in the fermentation process. *Penicillium* was absent in five fermented plant leaf hub networks, this indicated that the fermentation process clearly inhibited this taxon (**Figure 3**). The interactions between L and M communities also displayed a proportional balance of positive and negative connections. More than five keystone taxa were interacting with each other in the plant leaf hub network (**Figures 3E, F**). These results suggested that specific fungal species and their interactions play a crucial role in driving the fermentation of plant leaves, and these keystone taxa may have a significant impact on the regulation of plant leaf quality.

### Changes of metabolites and associated fungi during fermentation

**Figure 4:**
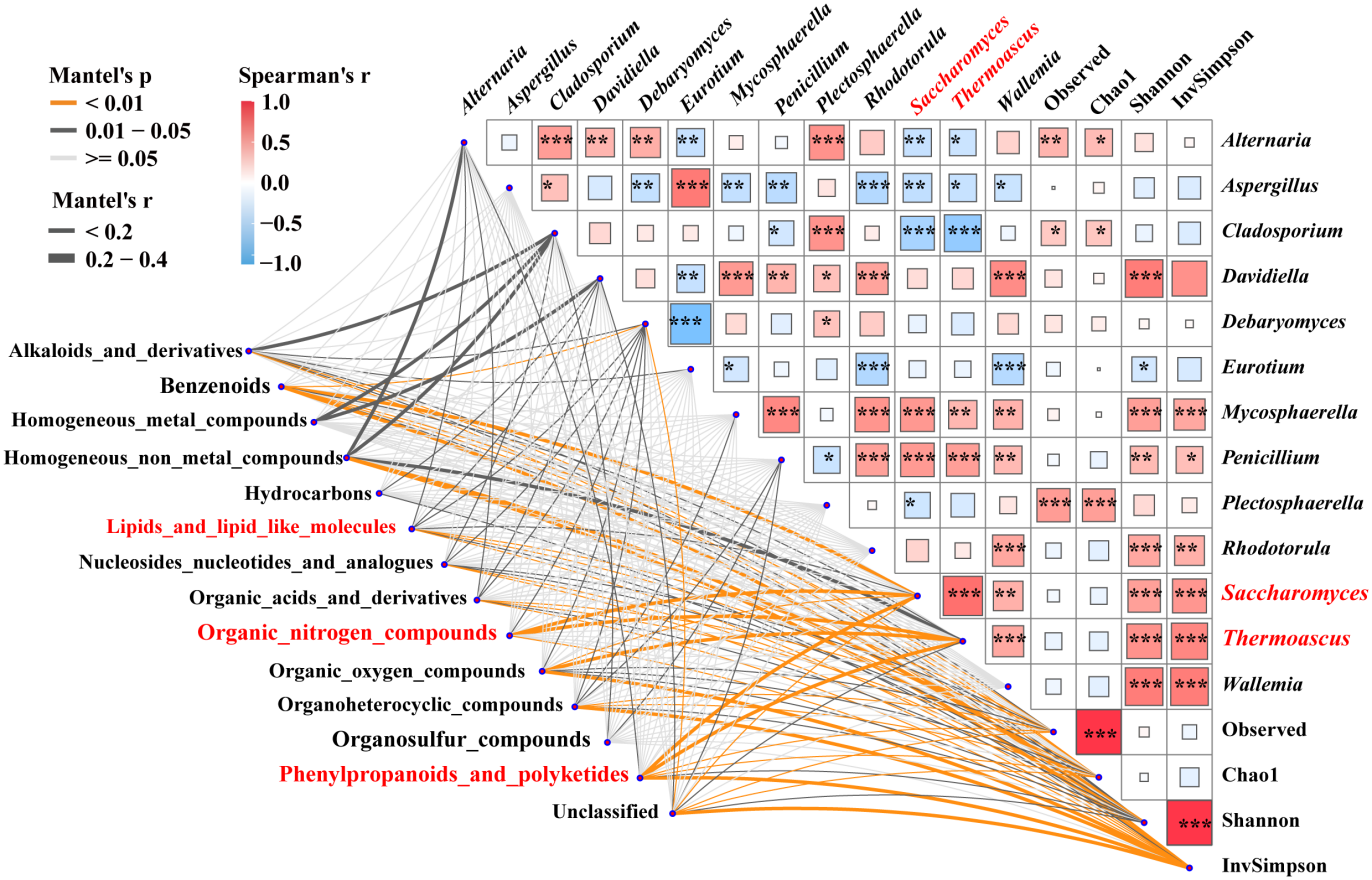
Correlations between metabolites and associated fungal diversity and keystones. Pairwise Spearman’s correlation matrix of the fungal community traits was shown with block charts, and metabolites with significant differences were related to each fungal diversity and biomarkers by Mantel tests during fermentation. Edge width means the Mantel’s statistic, and edge color means the statistical significance. *, **, *** represent *p* < 0.05, 0.01, 0.001, respectively.

Mantel tests were used to reveal the correlation between 3,460 specific metabolites, the fungal alpha-diversity, and 13 keystones (**Figure 4**). The contents of lipids, lipid molecules, organic nitrogen compounds, phenylpropanoids, and polyketides were significantly (*p* < 0.05) correlated with the relative abundance of *Saccharomyces*, respectively. Additionally, lipids and lipid-like molecules were significantly correlated with alpha-diversity and other biomarkers (e.g., *Alternaria*). Organic nitrogen compounds had an intensive correlation with the inverse Simpson index and the relative abundance of *Thermoascus* and *Debaryomyces*. Phenylpropanoids and polyketides were associated with alpha-diversity and the relative abundance of *Thermoascus* and *Alternaria*. The relative abundance of *Saccharomyces* had a strong positive correlation with the Shannon and inverse Simpson indices, as did the relative abundance of *Thermoascus*, *Mycosphaerella*, and *Penicillium*. Whereas, it had a strong negative correlation with the relative abundance of *Alternaria*, *Aspergillus*, and *Cladosporium*. These findings indicated that the contents of lipids, lipid-like molecules, organic nitrogen compounds, phenylpropanoids, polyketides, and the relative abundance of *Saccharomyces* were significantly correlated (*p <* 0.05). *Saccharomyces* was influenced by the interspecies interactions of these specific biomarkers.

**Figure 5:**
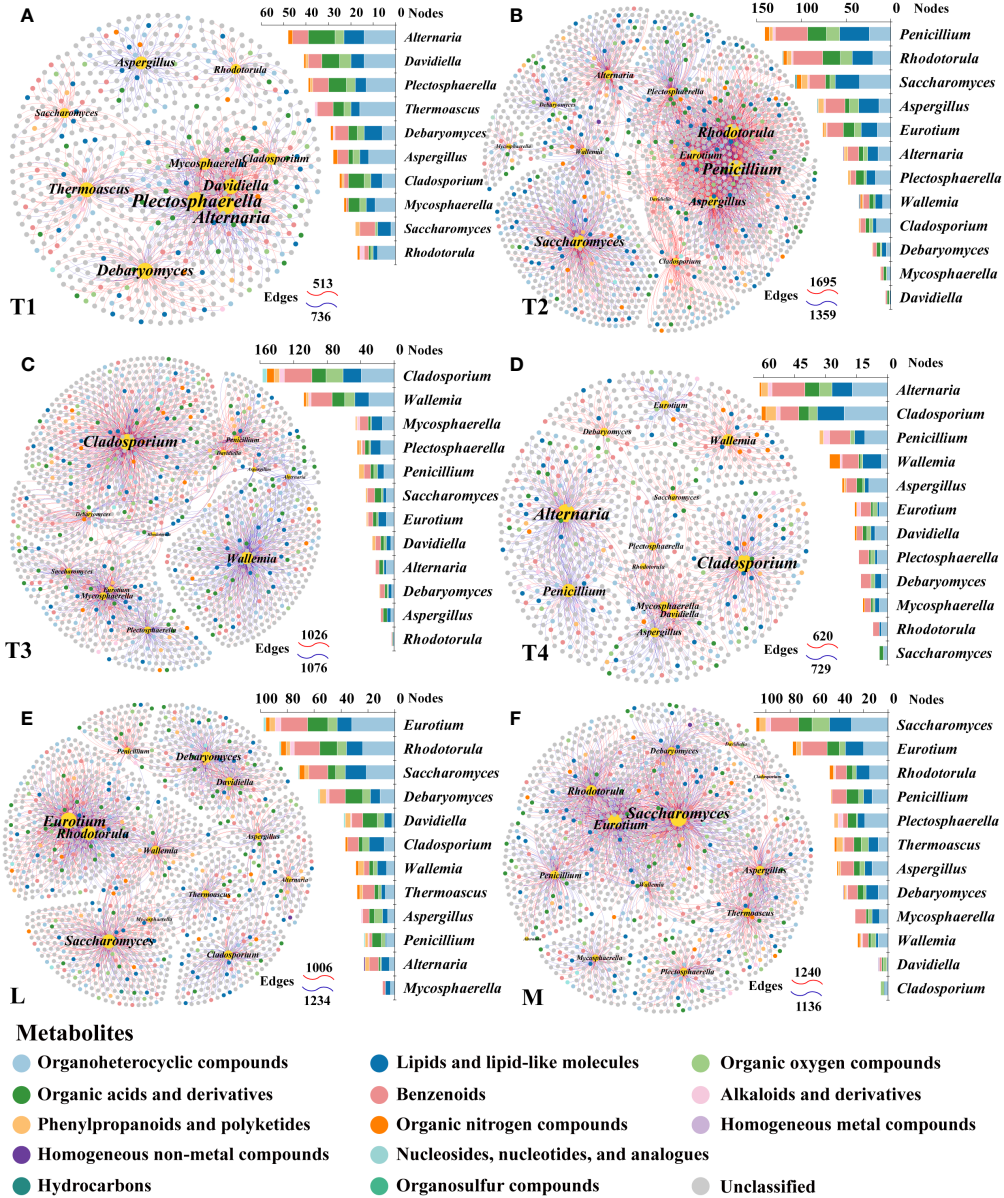
Correlations between metabolites and associated fungi in plant fermentation processing. Co-occurrence network (left) and the linked nodes number of key genera (right) under T1 **(A)**, T2 **(B)**, T3 **(C)**, T4 **(D)**, L **(E)**, and M **(F)**. In the co-occurrence network, the ginger nodes represent the degree of the OTUs, which belonged to 13 key genera that differentiate fermentation. The remaining nodes represent annotated metabolite ions, marked with different colors corresponding to the superclass level. A connection indicates a strong (Spearman’s |*r*| >0.7) and significant (*p* < 0.05) correlation. Positive correlations are shown by the red connections, while negative correlations are shown by the blue links. A node-edge statistics summary was provided, with numbers denoting edges.

Co-occurrence networks displayed distinct structure and topology for the correlations between 13 fungal biomarkers and variational metabolites among six groups (**Figure 5**). Generally, the co-networks were established by 13 keystones and 13 superclasses. The correlations were conspicuously dominated by different fungal species in a co-occurrence network of six groups, which indicated that specific fungal species and metabolites drive the fermentation process. *Saccharomyces* was the dominant genus in the M group, while *Eurotium* occupied the dominant position in the L group. The nodes of *Alternaria* had the highest linkages in T1 and T4. *Penicillium* and *Cladosporium* had the highest linkages in groups T2 and T3, respectively. The findings suggested that these genera may play critical roles in modulating the levels of harmful or beneficial metabolites, thereby influencing the quality of plant leaves (refer to **Figures 5E, F**). Additionally, there was a noticeable decline in network edges at the onset of fermentation. A rising trend was exhibited at the late fermentation stages (**Figures 5A–D**). In addition, the most abundant superclass was organoheterocyclic compounds in the six networks. Lipids and lipid-like molecules were the second dominant superclass, which exhibited higher abundance in the M than the L group. The linked edges of lipids and lipid-like molecules in fermented 8-day samples (T2) were also higher than in the other three fermentation time groups. These results suggested that lipids and lipid-like molecules significantly correlated with the quality of plant leaves during fermentation, and as this is changeful, it should be intently noticed in the early fermentation stage.

### Specific metabolites and fungi during fermentation

**Figure 6:**
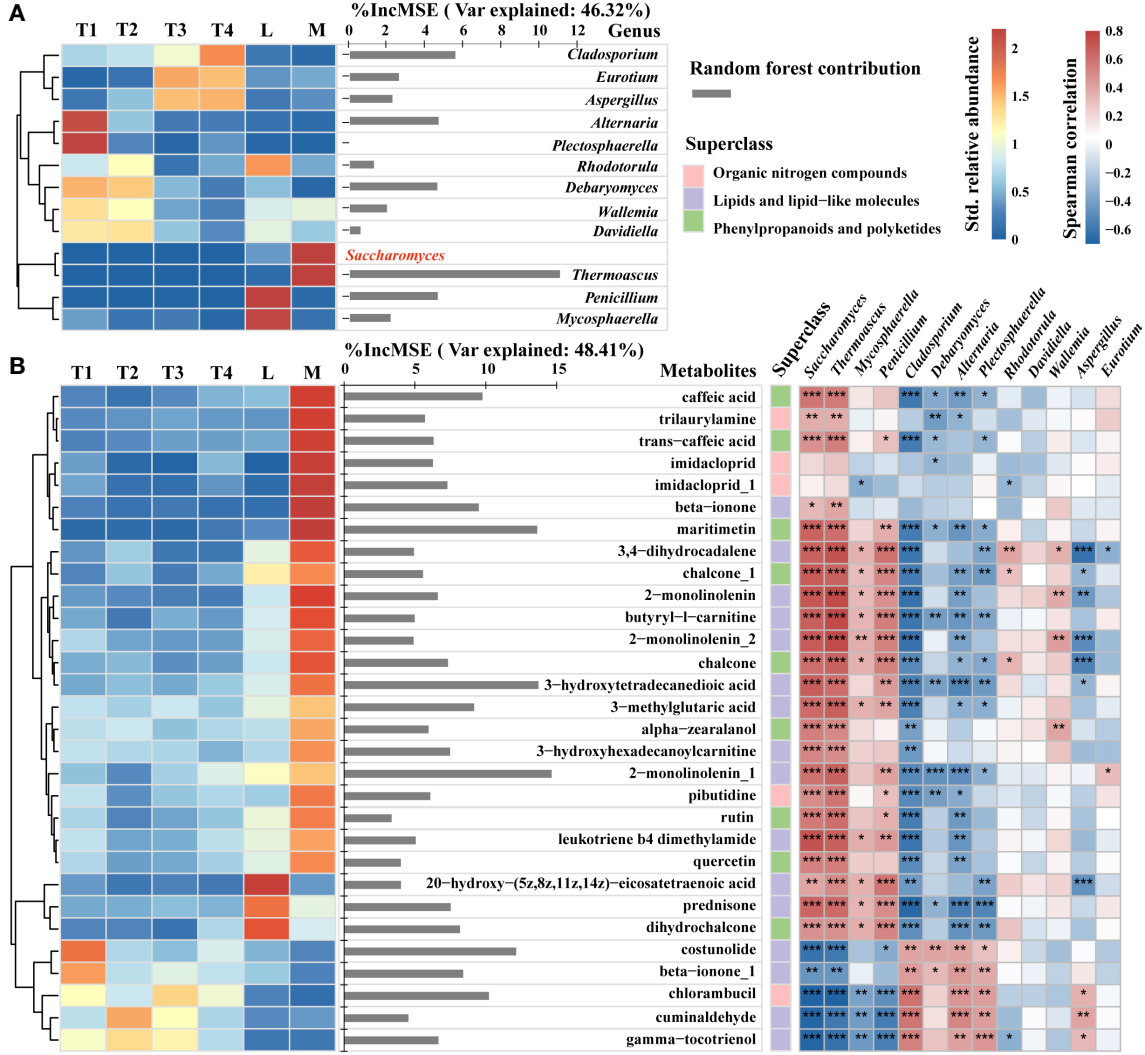
Contents and contribution of metabolite signatures and associated fungi of 12 key genera **(A)** and top 30 metabolite compounds **(B)** to *Saccharomyces*. Heatmap and hierarchical cluster analysis based on the standardized contents (left) and bar diagram (right) based on random forest contribution. The contents of certain taxa and metabolites increased with color. Red bars indicated positive associations, whereas blue bars indicated negative relations. The top 30 keystone metabolite predictors of *Saccharomyces* were found using random forest analysis. Each tree’s accuracy measure was calculated, and the results were averaged across the 2,000 trees in the forest. The significance of these predictors was calculated using the percentage increases in the mean squared error (MSE) of the variables; more significant predictors were indicated by larger MSE% values. *, **, *** represent *p* < 0.05, 0.01, 0.001, respectively.

To explore the changes in specific metabolites belonging to lipids, lipid-like molecules, organic nitrogen compounds, phenylpropanoids, and polyketides, and to reveal the relevance of specific metabolites and fungal species that were correlated with plant leaf quality enhancement, we conducted a specific analysis of the relative abundance of 13 key genera, the contents of pivotal metabolite compounds, and their contribution to *Saccharomyces.* A heatmap and hierarchical cluster analysis were used to intuitively reflect the differences among key fungal genera in six groups. The contribution of these 12 key genera to the relative abundance of *Saccharomyces* was 46.32%. *Thermoascus* had the highest contribution to *Saccharomyces* relative abundance. Additionally, *Cladosporium*, *Eurotium*, and *Aspergillus* are enriched in T3 and T4. *Alternaria* and *Plectosphaerella* were the most abundant in T1. *Saccharomyces* and *Thermoascus* in the M group had the highest relative abundance among the six groups. *Penicillium* and *Mycosphaerella* were abundant in L (**Figure 6A**).

The top 30 contributors were obtained from the metabolites of lipids, lipid-like molecules, organic nitrogen compounds, phenylpropanoids, and polyketides by random forest. Their contribution to the relative abundance of *Saccharomyces* was 48.41%. A total of 20 metabolites were enriched in the M group, and they had a significant (*p* < 0.05) positive correlation with the relative abundance of *Saccharomyces* (**Figure 6B**). Five metabolites, such as maritimetin, 3-hydroxytetradecanedioic acid, 2-monolinolenin, costunolide, and chlorambucil, had an above 10% increase in mean squared error, showing a remarkable influence to *Saccharomyces.* However, maritimetin belonged to phenylpropanoids and polyketides, whose content was highest in the M group and lower in the other five groups. 3-Hydroxytetradecanedioic acid and 2-monolinolenin belonged to lipids and lipid-like molecules, the contents of which were higher in the M than the L group. In four fermentation time groups, the content of 2-monolinolenin was increased in 8 fermented days (T2) and decreased in 16 and 24 days (T3 and T4). Moreover, we found that these three metabolites had a remarkably positive correlation with *Saccharomyces*, *Thermoascus*, and *Penicillium* (*p* < 0.01) and a strongly negative correlation with *Cladosporium*, *Debaryomyces*, *Alternaria*, and *Plectosphaerella* (*p* < 0.05). Costunolide and chlorambucil were classified under lipids, lipid-like molecules, and organic nitrogen compounds, respectively. Their content was highest in unfermented samples (T1) and lowest in high-quality samples (M). Furthermore, the content of costunolide decreased progressively over the course of fermentation. These findings indicated that *Saccharomyces*, as a key taxon, regulated beneficial metabolites to improve the quality of plant leaves. The former mentioned three metabolites and two genera (*Thermoascus* and *Penicillium*) that could enhance the relative abundance of *Saccharomyces.* In addition, the contents of costunolide and chlorambucil enriched in T1 with negative correlations to *Saccharomyces* indicated that the presence of these metabolites could potentially reduce the relative abundance of *Saccharomyces.*

## Discussion

Currently, plant leaf fermentation is being favored due to its greater controllability and efficiency (Liu F. et al., 2021). There is a growing interest in utilizing fermentation processes to enhance the quality and characteristics of plant leaves (Jia et al., 2023). Plant leaves possess specialized metabolites for flavor component production, and fungi have diverse effects on the production of these specialized metabolites during fermentation (Pusztahelyi et al., 2015; Zeng et al., 2019; Li J. et al., 2020). In this study, we evaluated the metabolites and fungal species present in different quality and fermented plant leaves. The most abundant distributions in the six groups were organoheterocyclic compounds, which could be significantly influenced during fermentation process. The contents of lipids, lipid-like molecules, organic nitrogen compounds, phenylpropanoids, and polyketides were identified as the main metabolic contributors to the high quality of plant leaves. The variations were observed in the composition and structure of the fungal community of plant leaves with different quality. *Alternaria* dominated in T1 and T4 groups; *Penicillium*, *Cladosporium*, *Eurotium*, and *Saccharomyces* were the most abundant genera in T2, T3, L, and M groups, respectively. In total, 13 genera (e.g., *Eurotium*, *Saccharomyces*) were identified as key biomarkers differentiating six groups during fermentation. These specific metabolites and fungal genera present promising avenues for further exploration in plant leaf fermentation processes.

Microorganisms can regulate metabolites and control the quality of plant leaves during fermentation (Shang et al., 2022). The highest relative abundance of *Saccharomyces*, amounting to 14.8%, was observed in high-quality plant samples. *Saccharomyces* emerged as a hub taxon in fungal interspecies interactions and was also the dominant node associated with the largest number of metabolites in the co-occurrence network. *Saccharomyces* is a probiotic yeast (Pais et al., 2020), and it is widely used as a cell factory for producing various products at high levels from different feedstocks (Lian et al., 2018). In winemaking, grape juice is inoculated with mixed cultures of *Saccharomyces*, which enhances acidity and improves the overall quality of wine in simultaneous and sequential co-fermentation (Gobbi et al., 2013). The “golden flowers” were found to be present in higher-priced tea commodities with superior product quality, such as Liupao tea mark, and *Eurotium* was shown to be the dominant genus in these “golden flowers” (Mao et al., 2017). According to Chen et al. (2023), *Eurotium* plays a significant role in the synthesis of the chemicals that give loose-leaf dark tea its distinct flavor and in providing a chemical foundation for quality control throughout the processing of dark tea. We found that *Eurotium* showed a considerable contribution with a significant negative correlation to *Saccharomyces.* The highest relative abundance of *Eurotium* (> 60%) was found in 16- and 24-day fermented samples (T3 and T4), respectively, indicating that the control of *Eurotium* was significantly correlated with the enhancement of fermented sample quality. We found that throughout the course of the 24-day fermentation period, there were notable changes in the metabolites and fungal markers detected in high-quality fermented plant leaf samples. It could indicate that variations in short-term fermentation markers can be used to forecast long-term fermentation outcomes, which in turn might help direct the industrial advancement of cigar fermentation. However, the fermentation conditions are not consistent with 75% vs. 62% humidity, and the changes in humidity can significantly influence microbial activities and metabolic processes during fermentation. Therefore, any predictive application of short-term markers to long-term fermentation should consider the specific conditions and be validated through additional studies.

The correlations between specialized metabolites and specific fungal species in plant leaves are responsible for flavor component production and quality improvement during fermentation. Jiang et al. (2023) demonstrated that lipids and lipid-like molecules are closely related to volatile metabolite formation. Li et al. (2022) revealed that black Huangjiu (a traditional alcoholic beverage) fermented by sequential inoculation with *Saccharomyces* had a taste that was stronger, sweeter, mellower, and softer. In this study, lipids and lipid-like molecules (e.g., 3-hydroxytetradecanedioic acid and 2-monolinolenin) were the most common in high-quality plant samples. The majority of contributors to lipids and lipid-like molecules exhibited a significant positive correlation with *Saccharomyces* (*p* < 0.05), a result that notably contrasts with the findings of Li et al. (2022). This may be correlated with the effect of correlations between specific fungal species and metabolites. Gobert et al. (2019) indicated that organic nitrogen is an essential nutrient and volatile compound for *Saccharomyces* during alcoholic fermentation. Here, we found chlorambucil, as one of the organic nitrogen compounds, had a strongly negative correlation with *Saccharomyces.* This indicated an increase in *Saccharomyces* with the consumption of chlorambucil in high-quality fermented leaves. Existing studies have demonstrated that phenylpropanoids and polyketides interacted with helpful soil microorganisms, encouraged secondary cell wall formation, and offered protection against a variety of plant diseases (Yu and Jez, 2008; Fiorito et al., 2019). Maritimetin belonged to phenylpropanoids and polyketides, which had a remarkably positive correlation with *Saccharomyces* (*p <* 0.05). This indicated that they may work together to contribute to the quality enhancement of plant leaves.

*Saccharomyces*, as a key taxon, can regulate specific fungal species and metabolites to improve the quality of plant leaves. Lipids, lipid-like molecules, organic nitrogen compounds, phenylpropanoids, and polyketides were plentifully distributed in high-quality plant samples; they have a significant (*p* < 0.05) correlation with *Saccharomyces.* However, our current understanding of plant fermentation is still incomplete. Furthermore, this study demonstrates the relevance of *Saccharomyces*, specific fungal species (e.g., *Eurotium* and *Thermoascus*), and certain metabolites (e.g., maritimetin, 3-hydroxytetradecanedioic acid, and 2-monolinolenin) to the quality of fermented plant leaves. The part resulting from the balanced interactions by *Saccharomyces* during the fermentation cannot be neglected in the initial stages of fermentation processing. However, the exact mechanism of how the lipids, lipid-like molecules, organic nitrogen compounds, phenylpropanoids, polyketides, and *Saccharomyces* improve fermented plant leaf quality still needs to be deeply explored.

In conclusion, we uncovered how crucial metabolites and fungal species drive the fermentation process to enhance the quality of plant leaves during fermentation and their changes in four time series fermentation groups (0, 8, 16, and 24 days). The results showed that organoheterocyclic was susceptibly affected in plant leaves during fermentation. It was reduced in high-quality samples and significantly changed after four fermentation times. Lipids, lipid-like molecules, organic nitrogen compounds, phenylpropanoids, and polyketides were most abundant in high-quality plant samples; they have a significant (*p* < 0.05) correlation with *Saccharomyces. Saccharomyces* (14.8%) was the dominant genus in high-quality samples, while *Eurotium* (> 60%) was abundant in 16- and 24-day fermentation samples. The quality of fermented leaves is improved by the correlations between these particular compounds and fungal species. A total of 22 metabolites were found to have a significant contribution to the relative abundance of *Saccharomyces.* The most significant metabolites among them were 2-monolinolenin, 3-hydroxytetradecanedioic acid, and maritimetin, which all had positive correlations with the relative abundance of *Saccharomyces.* These typical metabolites and fungi may be used for regulated and effective fermentation processes. This study may offer a useful and efficient experimental foundation for improving the quality of fermented plant leaves and optimizing the fermentation process.

## Data availability statement

The data presented in the study are deposited in the SRA database, accession number PRJNA1032646.

## Author contributions

LX: Data curation, Formal Analysis, Investigation, Writing – original draft. JSL: Investigation, Writing – original draft. JL: Data curation, Investigation, Writing – original draft. ZY: Data curation, Writing – original draft. ZC: Investigation, Writing – original draft. WC: Investigation, Writing – original draft. MZ: Formal Analysis, Investigation, Writing – original draft. DM: Writing – review & editing, Conceptualization, Supervision. YW: Writing – review & editing, Data curation, Visualization, Writing – original draft. HY: Conceptualization, Supervision, Writing – review & editing.
