# Supplementary material for: Enhancing the quality of fermented plant leaves: the role of metabolite signatures and associated fungi
Source: Front Plant Sci. 2024 Mar 20;15:1335850. doi: 10.3389/fpls.2024.1335850 (PMC10987691; doi:10.3389/fpls.2024.1335850)
Supplement: Supplementary file 2 [file DataSheet_1.pdf]

Supplementary Information

Metabolite signatures and associated fungi improve fermented plant leaves quality

Lei Xing[1 a b c], Jinshan Lei [a b c], Jie Liu [a b c], Zhen Yang [a b c], Zhishun Chai [a b c], Wen Cai [a b c], Min Zhang [d], Yujie Wang [d *] and Huaqun Yin [d e *]

[a] *China Tobacco Sichuan Industrial Co., Ltd, Chengdu, 610100, China*

[b] *Cigar Fermentation Technology Key Laboratory of China Tobacco (China Tobacco Sichuan Industrial Co.,Ltd.)*

[c] *Industrial Efficient Utilization of Domestic Cigar Tobacco Key Laboratory of Sichuan Province*

[d] *School of Minerals Processing and Bioengineering, Central South University, Changsha, 410083, China*

[e] *Key Laboratory of Biometallurgy, Ministry of Education, Changsha, 410083, China*

[*] Corresponding author: Yujie Wang, Huaqun Yin

E-mail address: yujie_W@163.com; yinhuaqun_cs@sina.com

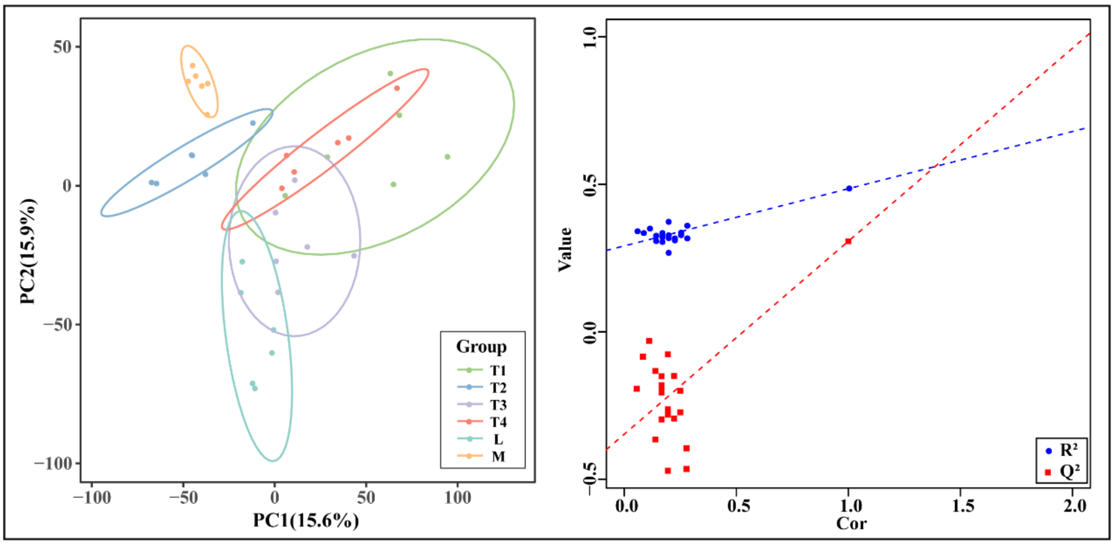

Fig S1. PLS-DA scores plot of differential metabolites.

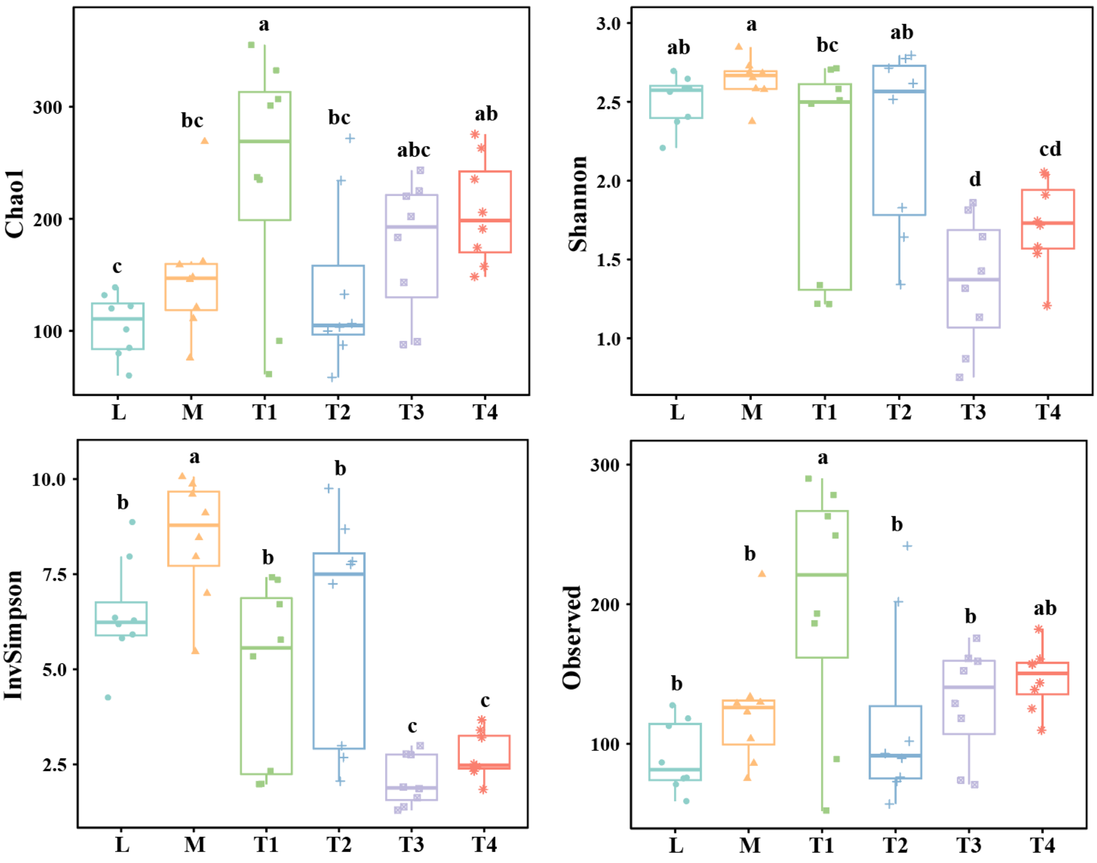

Fig S2. Alpha-diversity of cigar tobacco fungal communities. Different letters indicate significant difference between fermentation statuses ($P < 0.05$, ANOVA).

Table S1. Flavor components of the two cigar samples (L and M).

Indicators		L	M
Aroma characteristics	Bean scent	3	2
	Woody scent	2	1
	Burnt sweet	3	1
	Pure scent	0	1
	Smoky aroma	1	0
	Creamy	1	0
	Baked	2	0
Offensive odors	Scorched odor	0	1

(Score 0~5)

Table S2. Sensory characteristics of the two cigar samples (L and M).

Indicators		L	M
The mellowness of Aroma	Mellowness	6	5
	Richness	7	6
	Matureness	7	5
The mellowness of smoke	Plumpness	6	5
	Fluentness	6	5
	Smoothness	5	4
The refined purity of aftertaste	Sweetness	6	5
	Stimulating	6	6
	Cleanliness	6	6
	Aftertaste	5	5
Combustion characteristics	Combustibility	6	7
	Grey	6	6
	Ash coagulation	6	6
The harmonization of perceptible combustion and inhalation indices	Balancing	6.5	5

(Score 0~9)

Table S3. Statistical table of metabolite identification

mode	All	Metabolite	HMDB	KEGG
pos	15343	8451	2062	2396
neg	5103	2676	639	736

Table S4 Topological properties of cigar leaves community networks in different groups.

Network Indexes	T1	T2	T3	T4	L	M
Number of nodes	175	70	98	117	60	87
Number of edges	5117	352	638	1048	164	511
Avg. number of neighbors	58.480	10.057	13.020	17.915	5.467	11.747
Network diameter	4	6	5	5	6	4
Network radius	3	4	3	3	4	3
Characteristic path length	1.843	2.536	2.381	2.248	3.035	2.316
Clustering coefficient	0.702	0.509	0.501	0.498	0.406	0.442
Density	0.336	0.146	0.134	0.154	0.093	0.137
Heterogeneity	0.562	0.466	0.398	0.520	0.368	0.386
Centralization	0.305	0.163	0.095	0.202	0.079	0.122
Maximal degree	111	21	22	41	8	22
Nodes with max degree	OTU63(*Phaeosphaeriopsis*)	OTU42 (*Meyerozyma*)	OTU271 (*Cryptococcus*)	OTU116 (*Phaeosphaeria*)	OTU31 (*Wallemia*)	OTU30 (*Pleospora*)
